# Dielectric screening versus geometry deformation in two-dimensional allotropes of silicon and germanium

**DOI:** 10.1038/s41598-022-19260-y

**Published:** 2022-09-06

**Authors:** Antonello Sindona, Cristian Vacacela Gomez, Michele Pisarra

**Affiliations:** 1 Dipartimento di Fisica, Università della Calabria, Via P. Bucci, Cubo 30C, 87036 Rende, CS Italy; 2 INFN, Sezione LNF, Gruppo Collegato di Cosenza, Via P. Bucci, Cubo 31C, 87036 Rende, CS Italy; 3 Facultad de Ciencias, Escuela Superior Politécnica de Chimborazo (ESPOCH), 060155 Riobamba, Ecuador

**Keywords:** Condensed-matter physics, Electronic properties and materials, Graphene, Two-dimensional materials, Density functional theory, Method development

## Abstract

The search for connections between electronic and structural features is a key factor in the synthesis of artificial materials for on-demand applications, with graphene and analogous elemental semimetals playing a distinguished role as building blocks of photonic and plasmonic systems. In particular, a diversity of arrangements and electronic-state dispersions is offered by currently synthesized two-dimensional allotropes of silicon and germanium, respectively known as silicene and germanene. These monolayers make the ideal playground to understand how their collective and single-particle electronic states, excited by electron or light beams, may be controlled by geometry rather than doping or gating. Here, we provide such a study using time-dependent density-functional theory, in the random-phase approximation, to identify the structural dependent properties of charge-density plasmon oscillations and optical absorption in flat to buckled silicene and germanene lattices. We further single out flat germanene as an unprecedented two-dimensional conductor, hosting Dirac cone fermions in parallel with metal-like charge carriers, which contribute to strong intraband plasmon modes and one-electron excitations in the far-infrared limit. Finally, we show how this atypical scenario can be tuned by external stress or strain.

## Introduction

The isolation of graphene^1^, a monolayer of carbon atoms packed in honeycomb lattice, marked a turning point in material science leading to the synthesis of many other atomically thin crystals^2–4^, some of which can be vertically stacked on top of each other to form novel composite materials, held together by weak forces. Besides graphene itself^5^, the elemental analogues of graphene occupy a prominent position as building blocks of these so-called van der Waals (vdW) heterostructures^6^, having the potential of being assembled with on-demand geometries for specific electronic and optical purposes^7^.

On the theoretical side, buckled hexagonal phases of silicon and germanium atoms are predicted to exist in stable freestanding forms, respectively termed silicene and germanene^8–15^. Experimentally, differently buckled silicene and germanene monolayers have been epitaxially grown on top of crystal substrates^16–31^, presenting typical deformations, with respect to their freestanding geometry, due to the interlayer interaction and lattice mismatch. Some of these realizations, obtained on large band gap insulators and semiconductors, namely, h-BN^26–28^, AlN^29^ and MoS$_2$^30,31^, inherit most of the unprecedented mechanical, thermal and electrical properties of graphene, displaying in particular linear dispersing electronic states, or Dirac cone bands, around the Fermi energy.

If supplemented by further experiments and stability data, the above-mentioned silicene and germanene monolayers would make unique two-dimensional (2D) platforms compatible with current semiconductor manufacturing^32^. Furthermore, since silicon and germanium are heavier than carbon, they would present a larger spin-orbit coupling than graphene^33–36^, thus being better suited for spintronic applications. Hence, the major challenge is to link the outstanding electronic phenomena of the monolayers to their geometry, with attention to morphological changes caused by the interaction with the supporting substrate, the effect of another 2D material, e.g., in an heterostruture device, or even by an external tensile load (stress or strain).

Here, we scrutinize the dielectric properties of silicene and germanene, in flat to buckled hexagonal arrangements, with reference to graphene. We use a time-dependent (TD) density-functional theory (DFT) approach, in the random phase approximation (RPA), supported by an RPA kernel specifically designed for 2D materials. Accordingly, we compute the energy loss function and optical absorption of the intrinsic monolayers that, besides being accessible to experiments, allow us to explore the leading single-particle excitations (SPEs) and charge-density modes, or plasmon oscillations, activated by electron or light beams.

**Figure 1:**
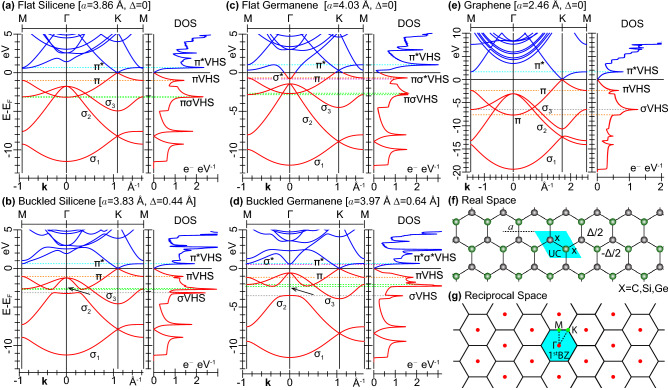
LDA band energies and densities of states of (**a**) flat silicene, (**b**) buckled silicene, (**c**) flat germanene, (**d**) buckled germanene, (**e**) graphene, along with the geometry information on the monolayers that include (**f**) real space lattice, unit-cell (UC), two-atom crystal basis (X–X), lattice constant *a*, buckling distance $\Delta$, (**g**) reciprocal lattice, 1st BZ, and M$\Gamma$KM contour. In all cases, the occupied bands and the lowest empty band have dominant $\sigma$, $\pi$ or $\sigma ^{*}$, $\pi ^{*}$ characters, with the three occupied $\sigma$ bands labelled $\sigma _1$–$\sigma _3$. The horizontal dashed lines mark the energy positions of the main VHS peaks, associated to the maxima or minima of these bands, at some eV from the Fermi level (set to zero energy, $E_F{=}0$). In particular, the $\pi ^{*}$VHS, $\pi$VHS, and $\sigma$VHS peaks present a well-defined, single-band character, whereas the $\sigma ^{*}\pi ^{*}$VHS, $\pi \sigma ^{*}$VHS, and $\pi \sigma$VHS structures arise from nearly degenerate maxima and minima of distinct bands with different characters. The black arrows point to the band gap around the $\pi \sigma$VHS structure, which opens up another $\sigma$VHS peak in buckled silicene and germanene.

Our primary analysis concerns the differences of the flat and freestanding buckled phases of silicene and germanene, whose main signatures appear in the large-momentum behavior of the visible (VIS) and ultraviolet (UV) plasmon modes. We further consider some intermediate buckled phases to better assess the link between the splitting of the VIS plasmon structure and the buckling level. Then, we show that some peculiar geometry conditions, occurring for example in flat germanene, may lead to new physics scenarios involving far-infrared (IR) to terahertz (THz) plasmons and intraband SPEs, suitable for photonic and electronic device technology. Finally, we monitor how this unprecedented condition changes with increasing the degree of buckling of the germanene monolayer.

## Results

### Electronic structure

**Figure 2:**
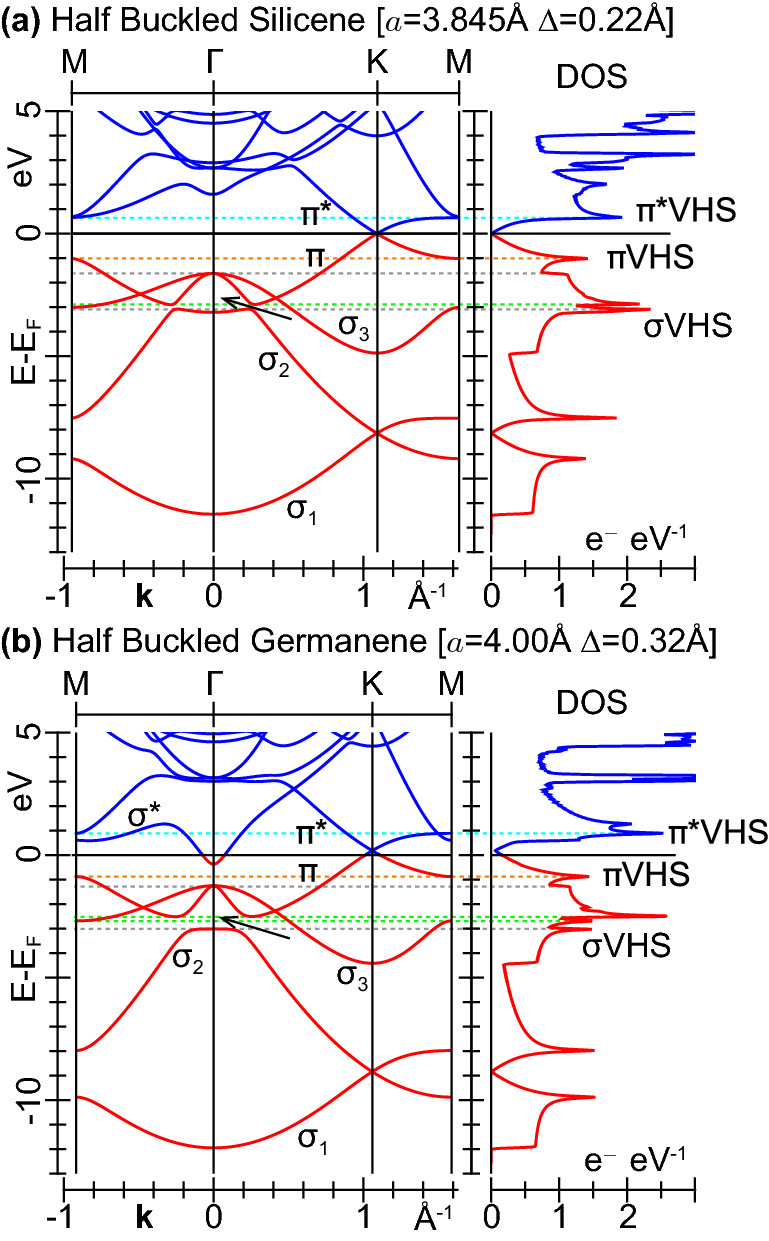
LDA band energies and densities of states of (**a**) half buckled silicene and (**b**) half buckled germanene, whose lattice constant *a* and buckling distance $\Delta$ are set to the average between the corresponding flat and freestanding buckled phases of Fig. 1a,b,c,d. All other details are as in Fig. 1.

To begin, we briefly discuss the band structure and density of states (DOS) of silicene and germanene, as compared to the more famous graphene^37^. Figures 1 and  2 summarize the results from our density functional calculations, which we performed using the plane-wave (PW) norm-conserving pseudopotential approach^38,39^, within the local density approximation (LDA), as detailed in "Density functional calculations" section.

Figures 1a–e and 2 show that all considered monolayers present three fully occupied bands of $\sigma$ symmetry, accordingly labelled $\sigma _1$–$\sigma _3$, in order of increasing energy. They are arranged in hexagonal lattice structures, set by the lattice constant *a*, with a crystal basis of two atoms per unit cell, as apparent in Fig. 1f. The latter contribute with eight valence electrons, as is typical of group-IV elements, yielding four completely filled bands. The highest occupied states can be of the $\pi$ or $\sigma ^{*}$ types, depending on the nature and geometry of the honeycomb lattice. Silicene and germanene further present an out-of-plane displacement of the atoms in the crystal basis, associated to the buckling distance $\Delta$. The $\pi$ and $\pi ^*$ bands exhibit linear energy-momentum dispersions, crossing at the six corners (K points) of the 1st BZ, reported in Fig. 1g, which give rise to the *well-known* Dirac cones.

Several Van Hove singularity (VHS) points can be spotted in the occupied and empty parts of the DOS profiles. These are also displayed in Figs. 1a–e and 2, being convolved with a Lorentzian lineshape having a phenomenological broadening of 0.01 eV. The two VHS peaks closer to the Fermi level $E_F$ are mostly associated to the $\pi$ and $\pi ^*$ bands, which approach the M point of the 1st BZ with flat dispersions.

At a closer look, flat silicene, see Fig. 1a, and graphene, see Fig. 1e, offer similar electronic features on different band widths. In particular, the Dirac cones of the two monolayers have slopes, or Fermi velocities, of $0.554{\times }10^{6}$ m/s and $0.829{\times }10^{6}$ m/s, respectively. The highest occupied $\sigma$ states and lowest unoccupied $\sigma ^{*}$ states are sufficiently far apart from the Dirac cone vertices. Then, the $\pi$ band is completely filled and the $\pi ^*$ band completely empty. This condition occurs at the charge neutrality point (or intrinsic state), which makes the two (undoped or ungated) systems zero-gap semimetals, with $E_F$ lying at the vertex of the Dirac cones, and the DOS vanishing at $E_F$. As another main distinctive feature, besides the band-width mismatch, the second lowest VHS structure in flat silicene arises from the $\pi$ band minimum at $\Gamma$ and the $\sigma _3$ band maximum at M, whereas in graphene it only comes the $\sigma _3$ band minimum at M.

We now recall that the most energetically stable geometry of freestanding silicene is a buckled honeycomb lattice^9^, whose band structure and DOS are shown in Fig. 1b. Upon comparison of Fig. 1a and Fig. 1b, we see that the Dirac cone features remain almost unaltered, with the Fermi velocity of the buckled phase being $0.542{\times }10^{6}$ m/s, that is ${\sim }98$% of the corresponding value in the flat phase, and ${\sim }65$% of the Fermi velocity of graphene. On the other hand, the broken in-plane mirror symmetry, induced by the buckling, opens a gap of ${\sim }0.4$ eV between the $\sigma _2$ and $\sigma _3$ bands, which in turns causes an avoided crossing between the $\pi$ and $\sigma _2$ bands, yielding distinct VHS peaks in the DOS at about $-3$ eV, relative to $E_F$.

Turning the focus to germanene with flat geometry, we notice that this monolayer exhibits unique electronic properties, which are absent in other x-enes. In particular, Fig. 1c shows that the lowest unoccupied band at $\Gamma$ has $\sigma ^*$ symmetry, being partially filled. As a consequence, the $\pi$ band gets partially empty, and the Dirac cone vertex is lifted above the Fermi level by ${\sim }0.3$  eV. The Dirac cone slope is $0.562{\times }10^{6}$ m/s, about ${\sim }68$% of the corresponding value in graphene. Inspecting the DOS, we find that it does not vanish at $E_F$, hence flat germanene is no longer a zero gap semimetal. Furthermore, the metal nature of flat germanene is atypical since two different bands (with different symmetries and energy-momentum dispersions) cross the Fermi level. This band crossing has also a minor effect on the highest occupied VHS peaks, which turns from $\pi$VHS to $\sigma ^{*}\pi$VHS.

Again, the most energetically stable planar allotrope of germanium form a buckled phase^9,10^. As reported in Fig. 1d, this freestanding germanene monolayer has a larger $\sigma _2$–$\pi$ gap opening, around 1.2 eV, than buckled silicene. In addition, the buckling restores the Fermi energy at the Dirac cone vertex, driving the bottom of $\sigma ^{*}$ band above $E_F$. Now, the $\sigma ^{*}$ band minimum at $\Gamma$ is nearly resonant with the $\pi ^{*}$ at M, which accordingly changes the nature of the lowest unoccupied VHS structure, from $\pi ^{*}$VHS to $\sigma ^{*}\pi ^{*}$VHS. The Fermi velocity of the sheet is $0.529{\times }10^{6}$ m/s, about 94% of the Dirac cone slope of the flat germanene phase, and 63% of the Fermi velocity in graphene. We further observe a direct band gap of ${\sim }1.15$ eV, between the degenerate $\sigma _3$, $\pi$ states and the $\sigma ^{*}$ states at $\Gamma$.

For control purposes, we also explore another configuration of silicene and germanene, with the defining parameters *a*, $\Delta$ taking intermediate values between the flat and freestanding buckled geometries. The electronic structures of the resulting ‘*half buckled*’ monolayers are given in Fig. 2. Here, we see that the band gap opening between the $\sigma _2$ and $\pi$ bands, reported for the freestanding buckled phases in Fig. 1b,d, is respectively lowered to ${\sim }0.2$  eV in half buckled silicene, as shown in Fig. 2a, and ${\sim }0.5$  eV in half buckled germanene, as shown in Fig. 2b. More importantly, the $\sigma ^{*}$ band of half buckled germanene still crosses the Fermi level at $\Gamma$, with the Dirac cone vertex lying at ${\sim }0.2$  eV above $E_F$, as also apparent in Fig. 2b, which gives this system the same atypical metal nature as flat germanene.

**Figure 3:**
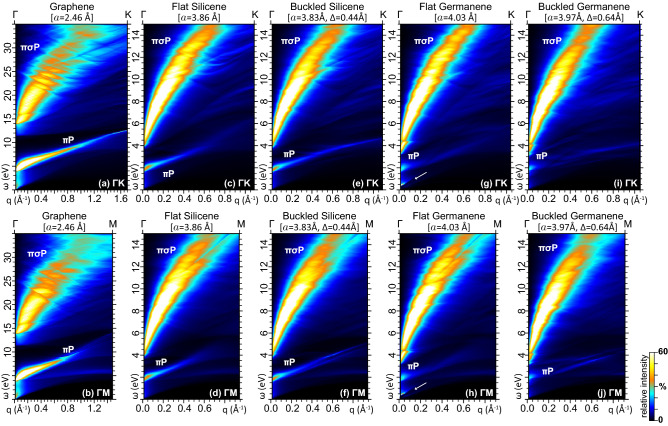
Energy Loss function of (**a**), (**b**) graphene, (**c**), (**d**) flat silicene, (**e**), (**f**) buckled silicene, (**g**), (**h**) flat germanene, (**i**), (**j**) buckled germanene for momentum transfers ${{\mathbf {q}}}$ along $\Gamma$K [top row: (a), (c), (e), (g), (i)] and $\Gamma$M [bottom row: (b), (d), (f), (h), (j)]. The intrinsic $\pi$ and $\pi \sigma$ plasmons, denoted $\pi$P and $\pi \sigma$P, produce distinct broad peaks in the VIS to UV band, propagating versus *q*. In flat germanene, another plasmon appears on the IR band, indicated by an arrow, which is due to the valence electrons populating the Dirac cone states, around K, and the $\sigma ^{*}$ states, around $\Gamma$. The intensity color scale in each density map is normalized to 60% of the maximum peak intensity.

The comparison of the flat, half buckled, and buckled phases of germanene proves that the energy position and dispersion of the two highest $\sigma _2$, $\sigma _3$ bands and the lowest $\sigma ^*$ bands can be modified near $\Gamma$ by induced stress or strain, as reported in previous studies^40,41^. Even more intriguing is the fact that buckled germanene, grown on specific substrates, presents the Dirac cones below $E_F$ at the K points, with partially empty $\sigma _2$, $\sigma _3$ bands, crossing $E_F$ around $\Gamma$^30,31^, which suggests investing additional effort in germanene-based assemblies.

### Energy loss spectra

The peculiar band dispersions and density of states illustrated above are key factors to the dielectric properties of the monolayers, as apparent from their macroscopic permittivity $\epsilon ^M$, which we computed by our specialized TDDFT approach, detailed in "Dielectric properties" section. Here, we focus on the energy loss (EL) function $E_{{\rm {loss}}}=-\mathrm {Im}(1/\epsilon ^M)$ for probing energies $\omega$, in the mid-IR to extreme-UV range, and momentum transfers ${{\mathbf {q}}}$, along the whole $\Gamma$K and $\Gamma$M paths of the 1st BZ, reported in Fig 1g. Besides being directly comparable with electron energy loss spectroscopy (EELS) experiments, such a quantity gives a detailed picture of plasmon propagation and damping.

To better understand the results, we recall the well characterized loss features of graphene^42–45^, computed for reference purposes and displayed in Fig. 3a,b. In the optical band ($\omega {\lesssim }3$ eV) and small-momentum region ($q{\lesssim }0.2$Å$^{-1}$), we detect a smooth background generated by SPEs around the Fermi level. At near- to far-UV energies ($\omega {\sim }4$–10 eV), we observe the most intense peak, which exhibits an approximate linear dispersion against *q*. This peak is usually ascribed to the $\pi$-plasmon (${\pi }$P), with the quantized charge-density mode being assisted by interband transitions between the $\pi$ and $\pi ^*$ bands, around the M point of the 1st BZ^42^. We further point out that momentum transfers $q>0.3$Å$^{-1}$, along $\Gamma$M, produce a splitting of the $\pi$P structure into different sub-peaks, with the lowest component exhibiting an almost flat ${\omega }{\,}vs{\,}q$ dispersion. Another very broad and less intense peak structure appears at far- to extreme-UV energies ($\omega {\gtrsim }12$ eV), which, at a closer inspection, exhibits different components, whose envelope have also an approximate linear ${\omega }{\,}vs{\,}q$ dispersion.

**Figure 4:**
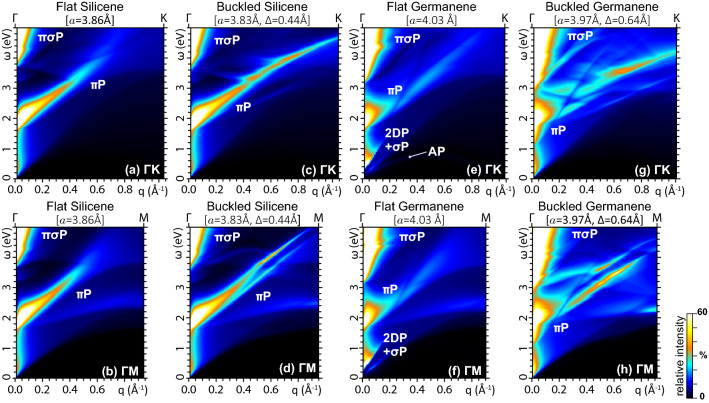
Energy Loss function of (**a**), (**b**) flat silicene, (**c**), (**d**) buckled silicene, (**e**), (**f**) flat germanene, and (**g**), (**h**) buckled germanene for $\omega <5$ eV and ${{\mathbf {q}}}$ along $\Gamma$K [top row: (a), (c), (e), (g)] and $\Gamma$M [bottom row: (b), (d), (f), (h)]. In flat germanene, the IR plasmon structure is resolved into its contribution from the Dirac cone electrons (2DP, AP) and the $\sigma ^{*}$ electrons. The intensity color scale in each density map is normalized to 60% of the $\pi$P peak intensity. The weak AP mode in (e) is barely visible with the current resolution and color scale settings. All other settings are as in Fig. 3.

This peak is usually ascribed to the $\pi \sigma$-plasmon (${\pi \sigma }$P), whose characteristic density wave is associated to interband transitions among the $\sigma$, $\pi$ and $\pi ^*$, $\sigma ^*$ bands^42^.

**Figure 5:**
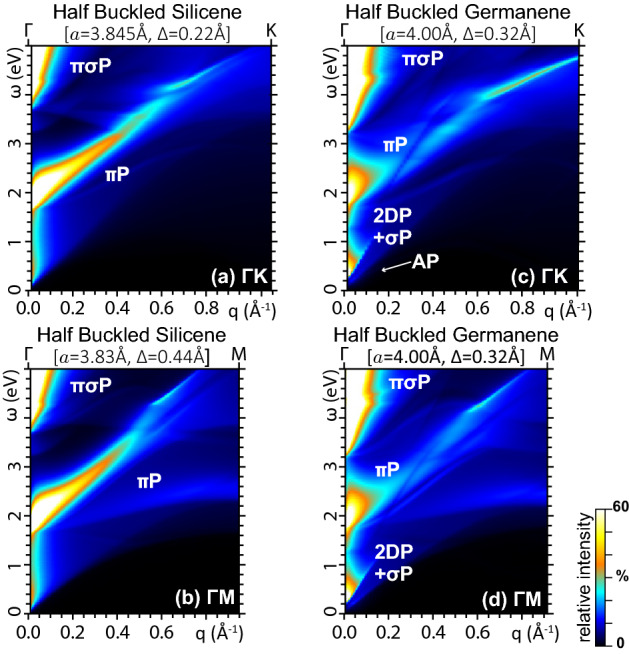
Energy Loss function of (**a**), (**b**) half buckled silicene, and (**c**), (**d**) half buckled germanene for $\omega <5$ eV and ${{\mathbf {q}}}$ along $\Gamma$K [top row: (a), (c)] and $\Gamma$M [bottom row: (b), (d)]. As in flat germanene, the IR plasmon structure of half buckled germanene is resolved into its contribution from the Dirac cone electrons (2DP, AP) and the $\sigma ^{*}$ electrons. All other settings are as in Fig. 4.

The energy loss properties of graphene guide our understanding of the EL spectra from the different phases of silicene and germanene, reported in Figs. 3c–j, 4, and 5. At first glance, we observe the same main features in all monolayers, namely, SP background, $\pi$P, and $\pi \sigma$P. Nonetheless, the energy extension of these structures, vertical axes in Figs. 3, 4, 5, depends on the widths of the highest occupied and lowest unoccupied bands.

Indeed, as discussed in "Electronic structure" section above, the band structures of silicene and germanene have the same nature as graphene, however the overall width of the occupied and empty bands implicated in the loss spectra is more than halved, with respect to graphene. At odds with what happens in graphene, the most intense feature in the EL function of silicene and germanene is the multiple-component ${\pi \sigma }$P structure. Upon comparing Fig. 3c,d with Fig. 3e,f, and, separately, Fig. 3g,h with Fig. 3i,j, we may conclude the following: as far as the ${\pi \sigma }$ plasmon is concerned, the flat and buckled phases of silicene and germanene present minor to negligible differences in the associated EL spectra; conversely, not only the $\pi$-plasmon dispersion, but also the near-UV to IR end of the EL spectra are highly dependent on the monolayer geometry; in particular, the hallmarks of an IR plasmon can be detected in the metal phase of flat germanene, due to both Dirac cone and $\sigma ^{*}$ electrons, with the electronic structure of Fig. 1c.

For a more in-depth appreciation of these differences, in Figs. 4 and 5 we zoom on the $\omega {<}5$ eV region of the EL spectra of silicene and germanene. The EL spectrum of flat silicene along $\Gamma$K, reported Fig. 4a, exhibits a well defined, linear dispersing $\pi$-plasmon peak. The same peak is also present along $\Gamma$M, see Fig. 4b, even though a new peak is detected, which exhibits an almost flat ${\omega }{\,}vs{\,}q$ dispersion for $q>0.4$Å$^{-1}$, in the same way as it happens in graphene. In buckled silicene, on the other hand, the main $\pi$-plasmon peak is split in, at least, two components along both $\Gamma$K and $\Gamma$M, as displayed in Fig. 4c and d. The peak splitting is as high as 0.5 eV, which is a signature of hybridization effects, induced by the buckling, between the $\pi$ and $\sigma$ bands of buckled silicene^46^. We may also infer that, the energy separation between the two components, the spectral weight of each peak, and the momentum range in which they appear, allow the double peak structure to be detected in high resolution (HR) EELS experiments.

The very peculiar band structure of flat germanene, shown in Fig. 1d, yields the most interesting features of the associated EL spectra for $\omega {\lesssim }5$ eV. Specifically, Fig. 4e and f show that the $\pi$-plasmon peak, starting at ${\sim }2$ eV in the optical momentum limit ($q{\ll }0.01$Å$^{-1}$), propagates with high intensity up momentum transfers around 0.2Å$^{-1}$. Then, as *q* further increases, the peak intensity sharply fades out, retaining however an almost linear ${\omega }{\,}vs{\,}q$ dispersion in its main component. A secondary component is more visible for ${{\mathbf {q}}}{\parallel }{\Gamma }M$, which ends up with an almost flat ${\omega }{\,}vs{\,}q$ dispersion as ${{\mathbf {q}}}$ approaches the border of the 1st BZ at M. Even more engaging are the different intense peaks found in the far-IR/THz and low momentum limit (three along $\Gamma$K and two along $\Gamma$M). These structures are caused by intraband plasmons generated by coherent charge oscillations in the partially filled $\sigma ^*$ band, and in the partially empty $\pi$ band, which are respectively associated to massive, parabolic-like charge carriers and massless Dirac cone fermions. We should now recall that charge carrier injection, e.g., by doping or gating, can activate the Dirac cone plasmons in graphene^47–51^ and silicene^46^. More specifically, extrinsic graphene and silicene exhibit a well-resolved and intense (in-phase, 2D) mode, exploitable for light confinement, and a weak (out-of-phase, acoustic) mode for momentum transfers along selected directions ($\mathbf { q}{\parallel }{\Gamma }K$).

The uniqueness of germanene sheets, also mentioned in "Electronic structure" section, is that even small geometry deformations, due to stress or strain^40,41^, or weak interaction with a supporting substrate^30,31^, can partially fill or empty the $\sigma$ or $\sigma ^{*}$ bands near the Fermi level, which is accordingly shifted below or above the Dirac cone. In these conditions, the 2D plasmon (2DP) is superimposed to a $\sigma$ plasmon ($\sigma$P), sharing similar intensities, while the acoustic mode is barely detected along $\Gamma$K.

Nonetheless, the most favourable geometry of freestanding germanene is the buckled phase of Fig. 1d, which leaves the $\sigma$ and $\sigma ^{*}$ states sufficiently below and above the Fermi level, respectively. In this semimetal phase, the germanene monolayer responds more similarly to graphene and silicene, as detailed in Fig. 4g and h. The intraband plasmon peaks are evidently absent. Again, the $\pi$-plasmon is split into different components. However, as in the case of the avoided crossing in the band structure, the splitting of the $\pi$-plasmon peak in buckled germanene is much more pronounced as compared to buckled silicene. We observe that, also in this case, the multiple components of the $\pi$-plasmon of buckled germanene are observable in HREELS measurements. Additionally, the SPE background is more intense than the other two semimetal monolayers.

To further clarify the picture, we explore the energy loss properties of half buckled silicene and half buckled germanene. In Fig. 5, we see that the splitting of the $\pi$P mode in multiple components is present in both monolayers, though it is less pronounced as compared to the full buckled geometries, which allow us to conclude that indeed this observable feature is a hallmark of the degree of buckling on the angstrom scale. Also clearly visible in Fig. 5c, d are the 2DP and $\sigma ^{*}$P modes of half buckled germanene, whose intensities appear to be comparable with those of the freestanding buckled phase, see Fig. 4e,f, because of the cutoff on the intensity color scale used in the associated density maps, which has allows us to better highlight the VIS-UV intrinsic plasmon structures. Nonetheless, this is a further evidence of how even small changes on germanene geometry can activate a strong interplay of the massless and massive plasmons.

### Optical absorption

We complete our study by analyzing the optical properties of our silicene and germanene monolayers, with reference to the well-known absorption response of graphene. We start by focussing on the macroscopic imaginary permittivity ${\mathrm {Im}}(\epsilon ^M)$ in the optical momentum limit, ${q}{\sim }0.0025$Å$^{-1}$, which provides the absorption transition rate as function of the excitation energy $\omega$. This quantity is comparable with absorption spectroscopy experiments, and provides complementary information relative to the EL function on the plasmon behavior versus $\omega$.

**Figure 6:**
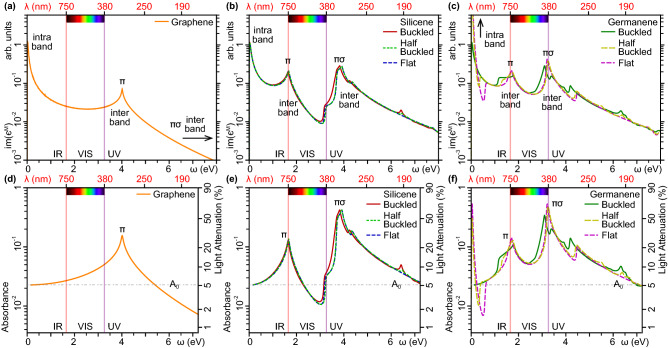
Optical absorption properties of (**a**), (**d**) graphene, (**b**), (**e**) flat, half buckled, and buckled silicene, (**c**), (**f**) flat, half buckled, and buckled germanene, expressed in terms of (**a**)–(**c**) the imaginary macroscopic permittivity ${\mathrm {Im}}(\epsilon ^M)$, and (**d**)–(**f**) the optical absorbance *A*. Both quantities are reported versus the excitation energy $\omega$ (lower horizontal axis) and the photon wavelength $\lambda$ (upper horizontal axis). In all cases, a fixed momentum transfer $q{\sim }0.0025$Å$^{-1}$ is applied along $\Gamma$M, which is the smallest value accessible in our 1$^\mathrm {st}$BZ sampling, and corresponds to the typical momentum of a photon in the few-eV energy range. In $\omega {\rightarrow }0$-limit, the optical absorbance of graphene, silicene, and buckled germanene tends to the same value $A_0=\pi \alpha$^52–55^, which is a fingerprint of the semimetal nature of the monolayers. On the other hand, the metal nature of flat and half buckled germanene corresponds to an infrared IR peak.

As suggested by Fig. 6a, the macroscopic imaginary permittivity of graphene is characterized by three peaks, the most intense of which appears in the THz limit ($\omega {\rightarrow }0$), accounting for low-energy interband transitions around the Fermi level. Then, in the IR to VIS range, ${\mathrm {Im}}(\epsilon ^M)$ follows a smooth profile, with a minimum intensity below 2% of the THz peak. The second-intense peak lies in the near-UV to mid-UV range, being generated by interband transitions between the $\pi$ and $\pi ^*$ states around the highest occupied and lowest unoccupied VHS points. This $\pi$ structure, found at ${\sim }4$ eV, presents a maximum intensity around 7% of the THz peak, and represents the optical ($q{\rightarrow }0$) counterpart of the $\pi$P feature in the loss spectrum of Fig. 3b. In the far- to extreme-UV range, $\mathrm {Im}(\epsilon ^M)$ drops down by several order of magnitudes. Nonetheless, a third- and least-intense peak is found at ${\omega }{\sim }12$ eV, above the energy range of Fig. 6a. This ${\pi }{\sigma }$ structure originates from interband transitions between the $\sigma$, $\pi$ and $\pi ^{*}$, $\sigma ^{*}$ states around the corresponding VHS points, see Fig. 1e. Accordingly, it represents the optical counterpart of the $\pi \sigma$P feature in the loss spectrum of Fig. 3b.

We observe the same three-peak structure in the macroscopic imaginary permittivity of silicene and germanene, either in flat, half buckled, or buckled geometry, as respectively reported in Fig. 6b and c.

Nonetheless, the narrower band width of the two monolayers, relative to graphene, makes these peaks appear at excitation energies $\omega <5$ eV, after which the intensity of ${\mathrm {Im}}(\epsilon ^M)$ drops down without noticeable features. In the case of silicene, we can hardly distinguish the differently buckled phases, with the only tiny exception of the onset of the $\pi \sigma$P structure, see Fig. 6b. In the case of germanene, on the other hand, the intensity of the THz peak scales inversely with the degree of buckling, being much higher in the flat and half buckled phases than in the buckled phase, see Fig. 6c. Additionally, the THz peak of the flat and half buckled phases is followed by a dip below 0.2 eV. This ${\omega }{\rightarrow }0$ feature is a clear hallmark of the above discussed metal-like band structure of germanene, shown in Figs. 1c and 2b, along with related intraband charge-density modes, given in Figs. 3g,h, 4e,f, and  5c,d.

The same structure in buckled germanene does not differ much from silicene. As for the $\pi$ structure, its peak position lies at $\sim 1.9$ eV, around the bottom limit of the visible spectrum, in both silicene and germanene, regardless of the buckling level. It is also worthwhile noticing that the $\pi$ peak of silicene, flat germanene, and half buckled germanene exhibits the same sharpness as the $\pi$ peak of graphene. In buckled germanene, however, the increased band gap opening around the $\pi \sigma$VHS, see arrow in Fig. 1d, produces broader lineshape, with a wider onset. As for the ${\pi \sigma }$ feature, it appears as a broad peak structure, mainly determined by transitions from the occupied $\pi \sigma$VHS states to the empty $\pi ^{*}$VHS states. In silicene, the main (most intense) component lies at $\sim 4$ eV, that is just outside the visible band. In germanene, on the other hand, the main peak is around $\sim 3.5$ eV, at the upper limit of the visible band. Also interesting is the fact that in flat and half buckled germanene, the main ${\pi }{\sigma }$ peak is very sharp, whereas in buckled germanene it is split into two components, which is also a consequence of the above mentioned band gap opening.

We now rely on the optical absorbance *A*, which provides a measure of the light intensity that is absorbed when photons of energy $\omega$ pass through graphene, see Fig. 6d, silicene, see Fig. 6e, and germanene, see Fig. 6f. Such a quantity, defined as the logarithmic ratio of transmitted to incident radiant power through the monolayers, is related to the imaginary macroscopic permittivity by^52,53^

1 $$\begin{aligned} A=\frac{\omega L}{c} \mathrm {Im}(\epsilon ^M) . \end{aligned}$$

Here, *c* is the speed of light, and *L* is the typical length of the system, which in our case coincides with the size of supercell in real space adopted for the calculation of $\epsilon ^M$. We now recall that the MP grids used for silicene, germanene ($720\times 720\times 1$) and graphene ($1200\times 120\times 1$) specify the 1st BZ samplings. These are respectively equivalent to construct a $720\times 720\times 1$ and $1200\times 1200\times 1$ supercell in real space, of size $L=720{\,}a$ and $L=1200{\,}a$, where *a* is the above mentioned lattice constant.

As a general feature, we observe that in graphene, silicene, and buckled germanene, the THz ($\omega {\rightarrow }0$) limit of the absorbance is $A{\rightarrow }A_0{=}\pi \alpha$, with $\alpha$ denoting the fine structure constant. Such a behavior is in perfect agreement with previous calculations^52–55^, and reflects an intrinsic property of Dirac cone semimetals^52^, where intraband transitions are practically absent. Again, the metal nature and correlated intraband plasmons of flat and half buckled germanene induce a very intense absorbance peak on the THz range, with $A{\rightarrow }0.53$, which corresponds to a light attenuation rate of $\sim 70$%, thus making the monolayer a potential 2D platform for optoelectronic applications.

At a closer view, Fig. 6d shows that graphene is mostly transparent for long wavelengths, from VIS to far-IR, where its absorbance has an average value of 0.02, i.e., an attenuance below 10%. The UV absorbance peak at $\sim 4$ eV corresponds to the $\pi$ peak of $\mathrm {Im}(\epsilon ^M)$, see Fig. 6a. At these photon energies, however, the attenuance is around 30%.

Turning to Fig. 6e, we still detect tiny differences in the absorbances of flat, half buckled and buckled silicene, as is the case of the imaginary macroscopic permittivity of Fig. 6b. In either geometry, the narrower valence band of silicene shifts the $\pi$ peak at the boundary between the IR and VIS bands, i.e., around $\omega {\sim }1.7$ eV. Nonetheless, the $\pi$ lineshape of graphene and silicene are similar in intensity and broadening. At VIS energies, the absorbance of silicene drops to a minimum near the boundary with the UV band, that is $\omega {\sim }3$ eV. Then, it increases up the $\pi \sigma$ peak, which occurs in the near UV range, at $\omega \sim 4$ eV. Around this photon energy the absorbance of silicene reaches its maximum $A{\sim }0.4$, equivalent to a light attenuation rate of ${\sim }60$%. We may, then, conclude that silicene is mostly transparent in the VIS band, with the exception of the lower end.

Finally, Fig. 6f shows that the absorbances of flat, half buckled and buckled germanene share similar values at energies above the near-IR range, whereas half buckled and flat germanene are characterized by the sharp intraband plasmon peak on the THz band. Indeed, the $\pi$ and $\pi \sigma$ structures of all phases appear very close in energy, though they display rather different widths and lineshapes. The $\pi$ absorbance reaches peak values of ${\sim }0.11$, in buckled germanene, ${\sim }0.13$, in half buckled germanene, and ${\sim }0.14$ in flat germanene, respectively equivalent to 23%, 26%, and 28% of light attenuation, at the IR-VIS boundary energy. The $\pi \sigma$ absorbance has maximum intensities of ${\sim }0.34$, with 54% of light attenuation in buckled germanene, ${\sim }0.48$ with 67% of light attenuation in half buckled germanene, and 0.53, with 70% of light attenuation in flat germanene, at the VIS-UV boundary energy. A common interesting feature is that the $\pi \sigma$ peak lies much closer to the $\pi$ peak with respect to silicene. As consequence, the VIS absorbance of germanene is generally larger than silicene and graphene, with attenuance rates always above 10%.

## Discussion

We have presented a comprehensive analysis of dielectric screening, electron energy loss and light absorption in silicene and germanene, with flat to buckled honeycomb lattice structures, in comparison with the analogous properties of graphene. Our primary focus has been on how the general features of the loss and absorption spectra are affected by geometry changes, with particular emphasis on the effect of the characteristic $\pi$ and $\pi \sigma$ plasmons that are commonly expected in group IV elemental monolayers.

We have singled out peculiar geometry conditions, e.g., flat and half buckled germanene, which may naturally host intraband plasmons excitations, due to charge carriers originating from both the partially empty Dirac cone states, below its vertex, and a bunch of parabolic dispersing states that cross the Fermi level. Our calculations suggest that the metal character of germanene, including the occupation of the $\sigma$ states and the shifting of the Dirac cone with respect to the Fermi level, increases with increasing the degree of flatness of the monolayer. This point is particularly appealing in view of some recently reported planar silicene structures^24,25^, which suggests directing more efforts towards the synthesis of flat germanene phases for applications to terahertz device technology.

Looking at the loss function, we have further observed that the splitting of the $\pi$-plasmon peak into multiple components with different intensities and dispersions is an observable signature of the flat and buckled geometries. In particular, buckled and half buckled silicene and germanene monolayers present two well separated sub-structures, which may be revealed in a HREELS experiment. As a general feature, we have found that the splitting of the $\pi$ plasmon structure is more evident and increases with increasing the degree of surface buckling. Accordingly, the presence or absence of the $\pi$ plasmon peak-splitting may be used as fingerprint to experimentally determine the degree of buckling of a silicene or germanene phase. In addition, we have well characterized the intraband plasmon response of flat germanene, being clearly distinguished form the SPE background recorded in all other monolayers.

On the optical side, we have spotted the main differences in the $\pi$ and $\sigma \pi$ excitation peaks, over the near-IR to far-UV band. Again, we have found an unprecedented response in the far-IR/THz absorption properties of flat germanene.

We therefore expect the presented framework to serve as a guide for the identification of the amount of buckling or flatness in a synthesized silicene or germanene structure, assessing the role of substrate influences, interlayer interactions, stress or strain conditions on the monolayer geometry.

## Methods

Our TDDFT approach was based on a package of Open-MP/MPI Fortran codes, developed by M.P. and A.S., which were interfaced with the DFT output from Abinit^38,39^, and implemented in one of the high-performance computing facilities provided by the CINECA consortium (Italy).

### Density functional calculations

As a starting point, we computed the ground state properties of silicene and germanene, in their flat to buckled hexagonal lattices, using the plane wave (PW) pseudopotential approach to Kohn-Sham (KS) DFT, as implemented in the Abinit package^38,39^.

We adopted the local density approximation (LDA), expressed in terms of the Teter-Pade exchange-correlation functional^56,57^ and a norm-conserving Troullier-Martins pseudopotential^58^. We further considered a cut-off of 50Ha, on the PW representation of the band-electron states, an energy convergence criterion of $10^{-12}$Ha, for self-consistent runs, and an out-of-plane vacuum region of $\sim$20Å. We carried out the First Brillouin zone (1st BZ) integrals on $\Gamma$-centered 90$\times$90$\times$1 Monkhorst-Pack (MP) grids. We took as reference some literature values for the geometry optimized lattice constant *a* and buckling distance $\Delta$, between the unit-cell atoms of the monolayers, to establish the flat and buckled phases^9^, which turned to be consistent with our LDA optimization. We specifically used $a=3.86$Å for flat silicene, $a=3.83$Å, $\Delta =0.44$Å for buckled silicene, $a=4.03$Å for flat germanene, and $a=3.97$Å, $\Delta =0.64$Å for buckled germanene.

We subsequently took the converged ground-state electron densities as inputs in non self consistent PW-DFT-LDA runs to obtain the KS electron energies $\varepsilon _{{\nu }{{\mathbf {k}}}}$ and wave functions $|{\nu }{{\mathbf {k}}}\rangle$, labelled by the band-index $\nu$ and wave vector ${{\mathbf {k}}}$, on highly refined MP grids. In particular, we employed $240\times 240\times 1$ MP grids, including ${\sim }64$ bands, to sample the energy region up to 20 eV above the Fermi level in our loss function calculations on silicene and germanene. We considered similar input parameters for the control calculations on graphene. With these settings, we generated the electronic bands and densities of states of Figs. 1 and 2. We applied even more refined MP grids to accurately compute the optical adsorption properties up to $\sim 10$eV, namely, $720\times 720\times 1$ for germanene and silicene, including more than 30 bands, and $1200\times 1200\times 1$ for graphene with at least 20 bands.

### Dielectric properties

With the ground state properties at hand, we first plugged the KS eigensystems $\{\varepsilon _{{\nu }{{\mathbf {k}}}},|{\nu }{{\mathbf {k}}}\rangle \}_{1^\mathrm {st}\mathrm {BZ}}$ in the Adler-Wiser formula^59,60^ to obtain the *non-interacting* density-density response function

2 $$\begin{aligned} \chi _{{{\mathbf {G}}} {{\mathbf {G}}}'}^{0}= \frac{2}{\Omega } \sum _{{{\mathbf {k}}},{\nu },{\nu '}} \frac{ (f_{{\nu }{{\mathbf {k}}}}-f_{{\nu '}{{\mathbf {k}}}+{{\mathbf {q}}}}) \rho _{\nu \nu '}^{{{\mathbf {k}}}{{\mathbf {q}}}}({{\mathbf {G}}})\, \rho _{\nu \nu '}^{{{\mathbf {k}}}{{\mathbf {q}}}}({{\mathbf {G}}}')^{*} }{\omega +\varepsilon _{{\nu }{{\mathbf {k}}}}-\varepsilon _{{\nu '}{{\mathbf {k}}}+{{\mathbf {q}}}}+\mathrm {i}\;\eta }, \end{aligned}$$

or *unperturbed* susceptibility, here reported in atomic units. In Eq. (2) above, $\omega$ is the energy and ${{\mathbf {q}}}$ the induced momentum of the external perturbation (photon or electron). $\eta$ is a positive infinitesimal broadening (hereinafter set to $\eta =0.01$eV, unless otherwise stated). $f_{\nu {{\mathbf {k}}}}$ and $f_{\nu ' {{\mathbf {k}}}+{{\mathbf {q}}}}$ are the Fermi-Dirac distribution factors, respectively, associated to the energy levels $\varepsilon _{\nu {{\mathbf {k}}}}$ and $\varepsilon _{\nu '{{\mathbf {k}}}+{{\mathbf {q}}}}$, whereas the spin degeneracy is taken into account by the factor of 2. $\rho _{\nu \nu '}^{{{\mathbf {k}}}{{\mathbf {q}}}}({{\mathbf {G}}})$ and $\rho _{\nu \nu '}^{{{\mathbf {k}}}{{\mathbf {q}}}}({{\mathbf {G}}}')^{*}$ respectively denote the screening matrix elements $\rho _{\nu \nu '}^{{{\mathbf {k}}}{{\mathbf {q}}}}({{\mathbf {G}}})=\langle \nu {{\mathbf {k}}}|e^{-\mathrm {i}({{\mathbf {q}}}+\mathbf { G})\cdot \mathbf {r}}|\nu ' {{\mathbf {k}}}+{{\mathbf {q}}}\rangle$ and $\rho _{\nu \nu '}^{{{\mathbf {k}}}{{\mathbf {q}}}}(\mathbf { G}')^{*}=\langle \nu ' {{\mathbf {k}}}+{{\mathbf {q}}}|e^{\mathrm {i}({{\mathbf {q}}}+{{\mathbf {G}}}')\cdot \mathbf {r}}|\nu {{\mathbf {k}}}\rangle$.

Second, we used $\chi ^{0}$ to obtain the interacting density-density response function, or full susceptibility $\chi$, through the fundamental equation of TDDFT^61,62^, having the matrix form

3 $$\begin{aligned} \chi _{{{\mathbf {G}}}{{\mathbf {G}}}'}= \chi ^0_{{{\mathbf {G}}}{{\mathbf {G}}}'} + (\chi ^0 v \chi )_{{{\mathbf {G}}}{{\mathbf {G}}}'}. \end{aligned}$$

In this, we employed the RPA, i.e., we neglected the exchange-correlation part of the kernel, and replaced *v* in Eq. (3) above by a Coulomb interaction. However, it is important to mention that the bare three-dimensional (3D) Coulomb potential, whose matrix elements in reciprocal space read $v^0_{{{\mathbf {G}}}{{\mathbf {G}}}'}=4\pi \delta _{{{\mathbf {G}}}{{\mathbf {G}}}'}/|{{\mathbf {q}}} + {{\mathbf {G}}}|^2$, is not appropriate for the description of the 2D materials in our specific approach. Indeed, the $v^0_{{{\mathbf {G}}}{{\mathbf {G}}}'}$ matrix elements would unphysically couple the infinitely periodic replicas of the honeycomb structures in the out of plane direction, no matter how far apart they are placed, in the PW-DFT approach outlined above. For these reasons, we adopted a well tested truncation scheme for the coulomb potential in the out-of-plane direction^43–45^, leading to

4 $$\begin{aligned} v_{{{\mathbf {G}}}{{\mathbf {G}}}'} =\int _{-L/2}^{L/2} dz \int _{-L/2}^{L/2} dz' \frac{2\pi e^{\mathrm {i} ({G_z} z-{G'_z} z')-|{{\mathbf {q}}} + \mathbf {g}||z - z'|}}{|{{\mathbf {q}}} + \mathbf {g}|}, \end{aligned}$$

with $\mathbf {g}$ and ${G_z}$, respectively, labelling the in-plane and out-of-plane components of ${{\mathbf {G}}}$.

The knowledge of $\chi$ gives access to the inverse dielectric matrix

5 $$\begin{aligned} \epsilon ^{-1}_{{{\mathbf {G}}} {{\mathbf {G}}}'}=\delta _{{{\mathbf {G}}}{{\mathbf {G}}}'}+(v \chi )_{{{\mathbf {G}}} {{\mathbf {G}}}'}, \end{aligned}$$

and the macroscopic dielectric function $\epsilon ^M=1/\epsilon ^{-1}_{{\boldsymbol{00}}}$. In all matrix quantities and equations above, we took into account of the *so-called* non-local field effect^63^, associated to the off-diagonal elements of $\chi ^0_{{{\mathbf {G}}}{{\mathbf {G}}}'}$, $\chi _{{{\mathbf {G}}}{{\mathbf {G}}}'}$, and $\epsilon _{{{\mathbf {G}}}{{\mathbf {G}}}'}$. We found well-converged results by selecting the smallest ${\sim }100$ ${{\mathbf {G}}}$-vectors of the form $(0,0,G_z)$. A similar selection scheme was used in previous works dealing with the dielectric response of graphene-based 2D systems^46–51^.

To clean off any thermal effects on the plasmons structure and related electron excitations, we performed all calculations with the statistical factors in Eq. (2), evaluated at zero temperature ($T=0$ K). We then obtained the EL function $E_{{\text {loss}}}=-\mathrm {Im}[(\epsilon ^{-1})_{\boldsymbol{00}}]$ by implementing Eqs. (2–5) on a wave vector grid of $240\times 240\times 1$ points, including all occupied and empty band energies up to ${\sim }20$ eV above $E_F$. With these settings, also reported in "Density functional calculations" Section, we computed the loss spectra of Figs. 3, 4, 5 within an energy resolution $\delta \omega$ of 0.01 eV, and an applied momentum resolution $\delta {q}$ of 0.0130Å$^{-1}$, along ${\Gamma }K$, and 0.0075Å$^{-1}$, along ${\Gamma }M$. As for the optical calculations, we computed $\mathrm {Im}(\epsilon ^M)$ and the absorbance of Eq. (1) by plugging the KS structure, refined on wave vector grids of $720\times 720\times 1$ points, for germanene and silicene, and $1200\times 1200\times 1$ points for graphene, in order to have an input momentum of ${q}{\sim }0.0025$Å$^{-1}$, along ${\Gamma }M$, which roughly matches the momentum of light at ${\sim }5$ eV. In doing so, we kept an energy resolution $\delta \omega {=}0.01$ eV in the spectra of Fig. 6.

## Data Availability

The authors declare that the data supporting the findings of this study are available within the paper. Further data, concerning the outputs and codes from DFT and TDDFT calculations, are also available from the corresponding author upon reasonable request.
